# How Porin Heterogeneity and Trade-Offs Affect the Antibiotic Susceptibility of Gram-Negative Bacteria

**DOI:** 10.3390/genes6041113

**Published:** 2015-10-21

**Authors:** Thomas Ferenci, Katherine Phan

**Affiliations:** School of Molecular Bioscience, University of Sydney, Sydney, NSW 2006, Australia; E-Mail: katherine.phan@sydney.edu.au

**Keywords:** bacterial diversity, trade-offs, nutrient uptake, outer membranes, antibiotic susceptibility

## Abstract

Variations in porin proteins are common in Gram-negative pathogens. Altered or absent porins reduce access of polar antibiotics across the outer membrane and can thus contribute to antibiotic resistance. Reduced permeability has a cost however, in lowering access to nutrients. This trade-off between permeability and nutritional competence is the source of considerable natural variation in porin gate-keeping. Mutational changes in this trade-off are frequently selected, so susceptibility to detergents and antibiotics is polymorphic in environmental isolates as well as pathogens. Understanding the mechanism, costs and heterogeneity of antibiotic exclusion by porins will be crucial in combating Gram negative infections.

## 1. Introduction

**Figure 1:**
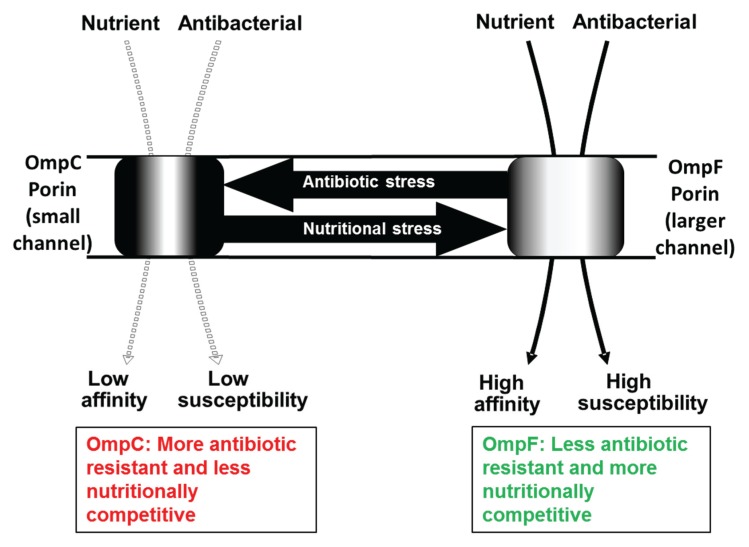
The major non-selective porins in the outer membrane of *E. coli*. OmpF porin permeates larger molecules at a faster rate and is induced by nutrient starvation. OmpC excludes larger molecules including antibiotics and detergents, so reduces susceptibility. OmpC is expressed instead of OmpF under stressful conditions.

Gram-negative bacteria are often poorly susceptible to antimicrobials. A contributing reason is that Gram-negatives have an intrinsic permeability barrier known as the outer membrane [1,2,3]. Hydrophilic solutes including many antibiotics cross the outer membrane through water-filled channels formed by a particular family of proteins known as porins. The permeability of polar solutes across the outer membrane of bacteria like *Escherichia coli* is controlled by the pore size of the porin proteins OmpF and OmpC, which are not specific for particular solutes. Optimal nutrient access is favoured by larger porin channels as in OmpF protein or additional, solute-selective proteins like LamB glycoporin in the outer membrane [4,5] or through several *Pseudomonas aeruginosa* porins like OprB and OprD [6,7]. High non-specific outer membrane permeability is a liability in unfavourable circumstances, such as with antibiotics. Access of toxic agents or detergents is generally minimised in *E. coli* through reduced outer membrane porosity and the increased proportion of smaller OmpC channels in the outer membrane [1,2,3]; see Figure 1.

Normally, the sum total amount of OmpF plus OmpC proteins is fairly constant in *E. coli*, but the relative proportion of the two varies subject to factors such as medium osmolarity, temperature, growth phase and the concentration of certain antibiotics in the medium [2,8,9,10]. Media with high osmolarity, high temperature or toxic ingredients favours the expression of OmpC and media of low osmolarity, low temperature and nutrient limitation increases OmpF and diminishes the level of OmpC [8,10]. The environmental integration of regulatory signals involves at least a dozen proteins in *E. coli* [11] and this complexity attests to the importance of getting correct settings of outer membrane permeability. From the antibiotic susceptibility perspective, the down-regulation of OmpF channels by MarA and Rob [11,12] is important as these are regulators of multiple antibiotic resistance. Less is known about the regulation of porins in other bacteria, but it is likely to be equally complex.

Under more extreme environmental conditions, strong selection is present for altering outer membrane permeability through mutations affecting porins. The extent of the native regulatory control of porin levels is insufficient to provide fitness under either nutrient starvation (when diffusion across the outer membrane becomes limiting) or when inhibitory molecules including antibiotics are present [13]. Structural and regulatory mutations affecting porins is particularly relevant, given they are part of the armory of emerging superbugs, particularly the multi-antimicrobial resistance problem in the treatment of Gram-negative bacterial infections due to Enterobacteriaceae and non-fermentative bacteria such as *Pseudomonas aeruginosa* and *Acinetobacter baumannii* [14]. Porins are also relevant to the emerging problems of resistance in *Enterobacter* [15] as well as *Neisseria gonorrhoeae* highly resistant to the expanded-spectrum cephalosporins [16].

In this communication, we consider the broader question of environmental situations when permeability either needs to decrease (e.g., with antibiotics) or increase (under extreme nutrient limitation). We also then discuss how the evolutionary constraints imposed by the sequence changes in porins leads to trade-offs between nutrition and resistance. We then discuss the inevitable heterogeneity imposed by a structural constraint trade-off, and how the shape of this trade-off affects bacterial diversity. Finally, the implications of porin heterogeneity in emerging antibiotic resistance are discussed.

## 2. Modifications Affecting Porins Influencing Antibiotic Susceptibility

In every Gram-negative bacterium investigated, two kinds of change relevant to porins are selected in mutationally acquired resistance. A common finding is a regulatory reduction in the quantity of porin, especially the larger-channel OmpF-like porins but sometimes all porins. A second mutational target is the structure of the porin protein itself, whose channel can be modified to change the structural or size-selectivity of the porin. These will be described in turn.

### 2.1. Regulatory Changes

Treatment with a number of antibiotics is associated with a heritable decrease in porin expression. For example, regulatory mutations in OmpR (a transcriptional controller of porin expression [17]) lead to the emergence of porin-deficient subpopulations with reduced carbapenem susceptibility in ESBL-producing *E. coli* [18]. Regulatory changes are a common response to treatment [15] and examples include *Klebsiella pneumoniae* and *Enterobacter aerogenes* with high-level ertapenem resistance lacking both of the major non-specific porins. Other examples include variable patterns of OmpC and OmpF expression in *E. cloacae* with lower-level ertapenem resistance [19]. OmpF and the EnvZ/OmpR proteins regulating porin expression are also important participants of the pathways regulating the nalidixic acid resistance of *E. coli* [20]. Cefoxitin resistance in *E. coli* is also associated with loss of both the OmpC and OmpF porin proteins and *Salmonella typhimurium* exposed to progressive increases in cephalosporin concentration exhibited mutations in regulatory genes (*envZ*, *cpxA*, or *nmpC*) that reduced expression of OmpC, OmpD, and OmpF porins [21]. Resistance in *Acinetobacter* species and *Pseudomonas aeruginosa* through diminished expression of porin is also common [22].

The associated costs of down-regulating porins are especially poorly understood. The absence of porins is deleterious in competing for nutrients as considered below, but further effects may be also involved. For example, mutations in porin regulation can involve regulatory protein like OmpR, RpoS, H-NS, RpoE, Lrp or small RNAs [11] that have a variety of other roles in the cell besides porin regulation. Mutations affecting these regulatory proteins may disrupt fitness in specific environments. Unexpected costs may apply to altered OmpR regulation, which is also involved in unrelated responses of *E. coli* such as to attack by the neutrophil bactericidal/permeability increasing protein [23]. Another functional role of porin is in virulence of *S. flexneri* [24] and *Salmonella* [25], which is affected in porin mutants. Such hidden costs are rarely considered or studied in acquired antibiotic resistance.

Down-regulation of porins has a multiplier effect in terms of antibiotic susceptibility because all polar antibiotics access the cell through porins. So reduction in OmpF may be selected with one antibiotic (e.g., chloramphenicol) but this also reduces susceptibility to structurally unrelated antibiotics (e.g., fluoroquinolones) [26]. It should be stressed that clinical resistance is always associated with additional resistance mechanisms besides porins, as in ESBL-producing *E. coli* [18] or various carbapenem resistant *K. pneumoniae* belonging to various clonal complexes [27,28]. Efflux is a particular important complementary contributor to exclusion-based antibiotic resistance and is often enhanced in combination with porin changes [29,30].

### 2.2. Structural Mutations

**Figure 2:**
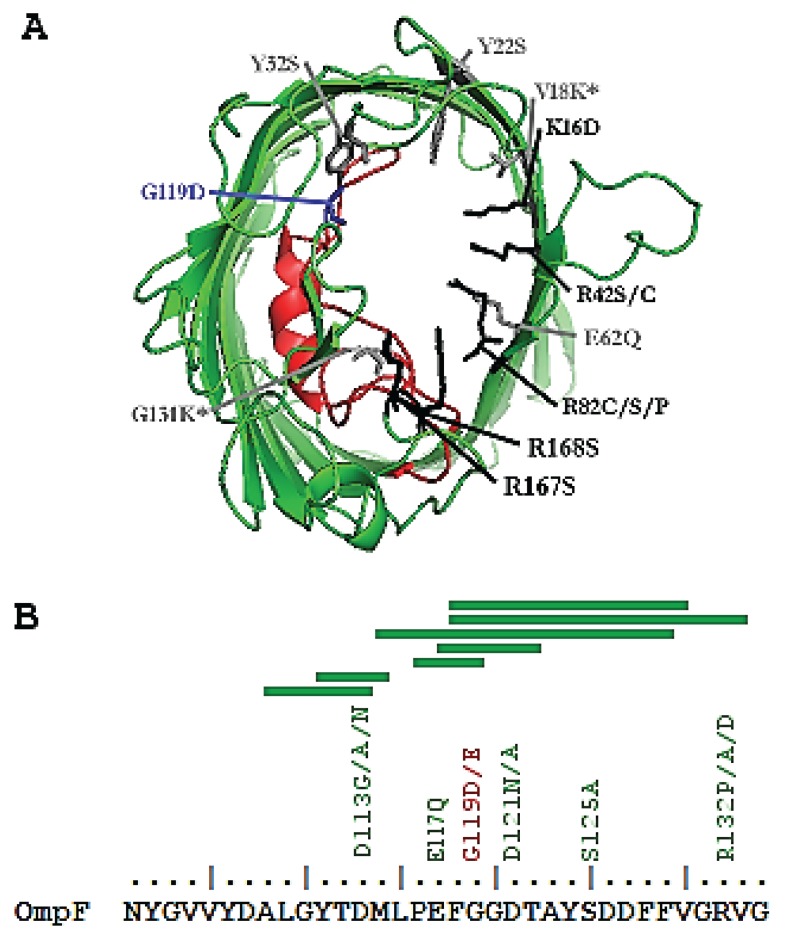
Structural mutations affecting the OmpF porin. (**A**) Cartoon diagram of the OmpF monomer within a trimer. The L3 constriction loop (red) and the sites of mutations increasing (black) or decreasing (grey) OmpF permeability are shown. Mutations marked with an asterisk occurred in conjunction. A mutation (G119D in *E. coli*) within the L3 loop (blue), is regularly observed in Gram negative organisms with decreased permeability. Adapted from PDB file 2OMF using PyMOL; (**B**) Amino acid sequence of the L3 loop in *E. coli* K-12 OmpF. Mutations increasing (green) and decreasing (red) permeability are shown. Areas of deletion in the L3 loop causing increased channel size are indicated by the green bars.

The detailed 3-dimensional structures of OmpF and C porins are known [31,32] and considerable information is available on the residues that affect permeability [33]. Both clinical and laboratory studies have identified a range of sites in both porin sequences that can either reduce or increase permeability of antibiotics through OmpF or OmpC. Examples of these are shown in Figure 2 and listed in Table 1.

**Table 1:** Documented changes in permeability in OmpF in Figure 2 with references.

OmpF Sequence Changes	Decreasing (D) or Increasing (I) Permeability	References
del116–119	I	[34]
del117–122	I	[34]
del108–114	I	[35,36]
del111–123	I	[37]
del114–129	I	[38]
del118–130	I	[39]
del118–133	I	[38]
Y22S	D	[40]
D121N/A	I	[40]
D113G/A/N	I	[33,34,36]
R132A/D/P	I	[33,35,41]
G119D/E	D	[42]
S125A	I	[40]
E117Q	I	[40]
R167S	I	[40]
R168S	I	[40]
R42S/C	I	[33,36,40]
K16D	I	[41]
R82C/S/P	I	[33,36,41]
E62Q	I	[40]
Y32S	I	[40]
V18K + G131K	D	[35]

Many of the structural changes in permeability affect the homologous L3 loop that protrudes into the water-filled channel at its constriction point in both porins, as illustrated in Figure 2A for OmpF. Other permeability changes are due to substitutions in residues lining the channel, often with side-chains facing the L3 loop as also shown in Figure 2A.

The L3 loop sequence is conserved between the *E. coli* OmpF and other porin sequences and Figure 2B) shows the conserved positions at which some of the permeability changes occur. The importance of the L3 loop in constricting the channel is also reinforced by the numerous deletions within the L3 sequence found in mutants selected for increased permeability. The kinds of selection conditions that give rise to raised permeability are discussed next.

### 2.3. Mutations Affecting Porins Selected under Nutrient Limitation Stress

It is important to remember that the main natural role of porins is the uptake of polar nutrients, including sugars, amino acids and ions [43]. The diffusion through water-filled channels is linearly dependent on the concentration gradient across the outer membrane so when ambient concentrations of external nutrients are low, permeability rates are limited by porin function. There is thus a strong, consistent selection pressure in the absence of antibiotics to improve permeability rates, which is simple to achieve by mutational changes affecting porins. Continued growth under nutrient limitation results in mutationally elevated porin levels [34,44] and structural mutation in OmpF in the L3 loop (including some of the short deletions In Figure 2b) are also a solution to the problem [34]. Porins also exhibit exclusion of larger polar molecules that do not fit the channel and growth selection in the presence of maltooligosaccharide sugars containing >3 glucose residues also selects for increased channel size [36]. The sites of mutations resulting in increased permeability for dextrins overlap in L3 with those selected under nutrient limitation in Figure 2A,B. A physiological and clinical consequence of these up-permeability changes is that the bacteria exhibiting these changes show greater susceptibility to antibiotics that can now cross the larger channels with ease.

## 3. Porins as Perfect Examples of a Design Constraint Trade-Off

As is amply evident from the above properties, there is a negative correlation between nutrient permeability and the resistance characteristics of porins; this is the essence of a trade-off between two traits. Trade-offs rule out that both traits are optimized at the same time so the optimisation of one cellular property occurs at the expense of another. It is a consequence of the constraint that larger channels are more generally permeable that causes the trade-off, and this is known as a structural constraint trade-off.

**Figure 3:**
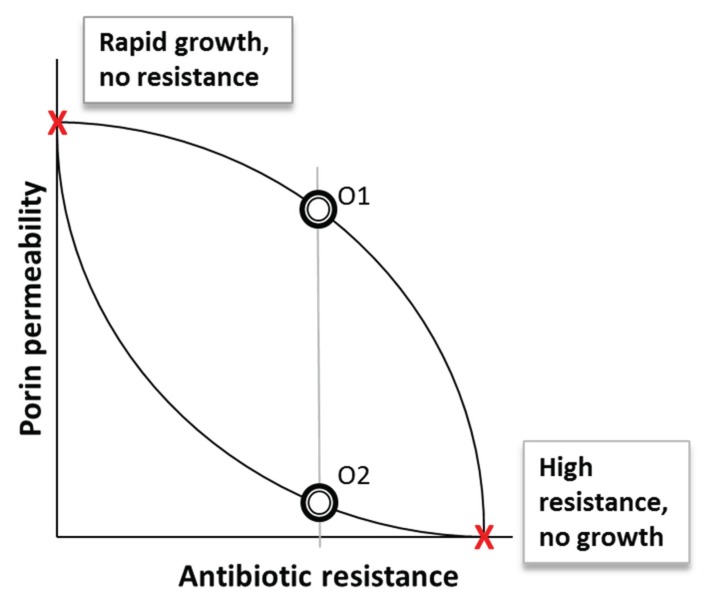
Trade-off shapes determining the behaviour of bacteria. Porins in *E. coli* have evolved to be neither specialist, highly permeable channels nor specialists in excluding everything including nutrients (red symbols). Most porins show intermediate, more generalist properties (circles) which may fall on different lines between the two extremes (called different trade-off shapes). Organisms O1 and O2 falling on different trade-off shapes can have very different properties. In this example, O1 is more generally fit in having the best combination of growth and resistance. The actual curvature of trade-offs is unknown for most systems, the shapes are being investigated for *E. coli* [47,48].

It is not generally appreciated by microbiologists that the shape of a trade-off is not necessarily linear between two negatively correlated characteristics and the curvature shown by intermediate settings of the trade-off has long been known to influence coexistence and heterogeneity in a species [45,46]. As shown in Figure 3, most porins are not specialized on either resistance or growth. As shown with organism O1, this intermediate setting falls on a concave curve which allows the emergence of generalists; in the case of porins, this would allow the emergence of the type shown which would have both appreciable resistance AND ability to trap nutrients in an intermediate setting. In contrast, the trade-off shape associated with organism O2 would predict that intermediate settings are unlikely to be fit in both dimensions because a small increase in fitness also leads to a rapid drop in resistance. In order to understand the emergence of porin mutations with altered trade-off settings, including those that contribute to antibiotic resistance, an understanding of the fitness costs associated with these trade-off shapes is an important need. The actual trade-off shapes are unknown for most systems including porins, but we need to determine the shapes to understand how bacteria evolve towards greater resistance. A means of studying these shapes was reported recently [47].

## 4. Bacterial Heterogeneity in Porins as a Solution to Alternating Environments

**Figure 4:**
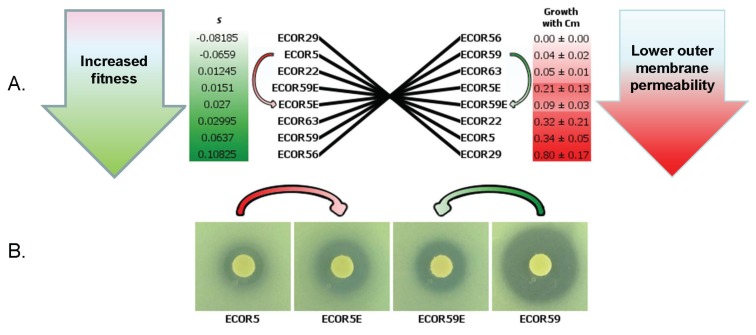
(**A**) reciprocal, variable trade-off between fitness and susceptibility evident in natural *E. coli* isolates. Fitter strains are more susceptible to antibiotics; for example, ECOR29 is the least fit but the most resistant. Conversely, ECOR56 is the fittest, but also the most susceptible. The growth of the *E. coli* ECOR strain set on plates containing 3 μg/mL chloramphenicol (Cm) was determined as in [50]. Fitness measured as a selection co-efficient (s) was obtained after 48 h of growth in competition with a reference strain (MG1655) in nutrient-limited chemostats from data in [50]. Mutational shifts in SPANC setting are shown by arrows for strains evolved after nutrient limitation (ECOR5E) or growth in the presence of antibiotic (ECOR59E); (**B**) Changes in the zone of inhibition of ECOR and derived strains were determined using disks with 8 μg/mL Cm.

Given that there is no optimal setting for permeability under all environmental conditions, it is perhaps not surprising that natural isolates including clinical isolates are found that have permeability properties that were selected in the recent history of the bacteria. Indeed, a comparison of 72 natural isolates (The ECOR set of *E. colis* [49]) indicated that antibiotic susceptibility and growth fitness under nutrient limitation were variable and formed a continuum of these phenotypes across the species [50]. It was evident that an inverse relationship between competitiveness and the resistance of strains to detergent and antibiotic was observable in species *E. coli* (Figure 4A). This is entirely consistent with the notion that membrane permeability and competitive fitness are linked by a trade-off between self-preservation and nutritional competence (SPANC; [13]); high permeability has a postulated cost in antibacterial sensitivity whereas a low permeability has a cost in nutrient affinity. This in turn means that scavenging ability is subject to trade-offs that limit competitive ability in individuals, but not necessarily the species as a whole. The extensive diversity in competitive characteristics we find under nutrient limitation within a bacterial species suggests that high nutrient affinity (large porin channels) carries a cost in some environments.

The continuum found also suggests that the trade-off is constantly reasserting, and this can be demonstrated in the laboratory. A strain which is less fit but more resistant can rapidly mutate in a nutrient limited environment to be fitter but less resistant, as shown in Figure 4B. Conversely, a strain which is fit but less susceptible can acquire mutations that make the strain more resistant in the presence of antibiotic, but less fit. Antibiotic susceptibility can frequently change in nature and these results confirm the flexibility of trade-off settings and the diversity shown in nature [50]. In addition, levels of porins may be subject to stochastic variation, permitting sub-populations to “adapt” by bet-hedging [51], but this remains to be investigated.

## 5. Conclusions on the Role of Porins in Antibiotic Resistance

The selection of porin mutations, as with all drug resistance, is best understood in the context of fitness in the host environment [52]. When bombarded with antibiotic, the hidden cost of reduction of porin permeability may be irrelevant in some sites in a patient but still carry a cost in another organ or environment. An example is that an OmpR mutation is a particular problem for *E. coli* in the urinary tract [53]. The overall survival a clone is thus not measurable as a single fitness in laboratory environments or even in different sites within a patient. For example, the cost of porin deficiency will be minimal at saturating nutrient levels in rich media, because a high nutrient gradient across the outer membrane is sufficient to supply nutrient even with a mutant channel. On the other hand, the cost may be high in nutrient limited sites. These actual costs of the trade-off and changing trade-off settings need to be studied, especially within the context of trade-off shapes that govern the interaction between traits such as resistance and nutrition. The heterogeneities in porins will become even more apparent when genome sequences emerge through deep sequencing of resistant isolates and evolution experiments [54,55]. Future studies of porins will thus aid the understanding of antibiotic susceptibility, the emergence of antibiotic resistance, and underpin antibacterial screening and design [56].
